# Toxicity study on *Clinacanthus nutans* leaf hexane fraction using *Danio rerio* embryos

**DOI:** 10.1016/j.toxrep.2019.10.020

**Published:** 2019-10-25

**Authors:** Suganya Murugesu, Alfi Khatib, Qamar Uddin Ahmed, Zalikha Ibrahim, Bisha Fathamah Uzir, Khaled Benchoula, Nik Idris Nik Yusoff, Vikneswari Perumal, Mohamed F. Alajmi, Sahal Salamah, Hesham R. El-Seedi

**Affiliations:** a Pharmacognosy Research Group, Department of Pharmaceutical Chemistry, Kulliyyah of Pharmacy, International Islamic University Malaysia, Kuantan, Pahang Darul Makmur, Malaysia; b Faculty of Pharmacy & Health Sciences, Universiti Kuala Lumpur Royal College of Medicine Perak, 30450 Ipoh, Perak Darul Ridzuan, Malaysia; c Central Research and Animal Facility, Kulliyyah of Science, International Islamic, University Malaysia, Kuantan, 25200 Pahang Darul Makmur, Malaysia; d Faculty of Pharmacy, Airlangga University, Surabaya, 60155, Indonesia; e Department of Pharmacognosy, College of Pharmacy, King Saud University, Riyadh, 11451, Saudi Arabia; f King Saud bin Abdul-Aziz University for Health Sciences, Jeddah 21423, Saudi Arabia; g Al-Rayan Research and Innovation Center, Al-Rayan Colleges, Medina, 42541, Saudi Arabia; h Pharmacognosy Group, Department of Medicinal Chemistry, Biomedical Centre, Uppsala University, Box 574, SE-751 23, Uppsala, Sweden; i International Research Center for Food Nutrition and Safety, Jiangsu University, Zhenjiang 212013, China

**Keywords:** *Clinacanthus nutans*, *Danio rerio*, DanioScope, Median lethal concentration, Teratogenicity, Probit analysis

## Abstract

*Clinacanthus nutans*, an herbal shrub belonging to the Acanthaceae family, is traditionally used as a functional food to treat various ailments in Malaysia and Indonesia. Although the polar fraction of this plant shows non-toxic effect, the toxicity of the non-polar extract is not reported so far. The present study aimed to assess the toxic effect and determine the lethal concentration of this non-polar fraction using zebrafish embryos. The *n-*hexane fraction was partitioned from the crude extract of *C. nutans* obtained using 80% methanolic solution. After spawning of the adult male and female zebrafish, the eggs were collected, transferred into a 96-well plate and incubated with the *n-*hexane fraction at concentrations of 15.63 μg/ml, 31.25 μg/ml, 62.5 μg/ml, 125 μg/ml, 250 μg/ml and 500 μg/ml in 2% DMSO. The survival and sublethal endpoint were assessed, the mortality and hatchability rates were calculated based on microscopic observation, while the heartbeat rate was measured using DanioScope software. The median lethal concentration (LC_50_) of the *C. nutans n-*hexane fraction, which was determined using probit analysis, was calculated to be 75.49 μg/mL, which is harmful. Moreover, gas chromatography-mass spectrometry (GC–MS) analysis revealed the presence of palmitic acid, phytol, hexadecanoic acid, 1-monopalmitin, stigmast-5-ene, pentadecanoic acid, heptadecanoic acid, 1-linolenoylglycerol and stigmasterol in the *n*-hexane fraction.

## Highlights

• The *C. nutans n*-hexane fraction is harmful with the LC_50_ of 75.49 μg/mL.
• The mortality rate increased as the fraction concentration increased.
• Morphological defects such as less pigmentation, dented tail, oedema malformed yolk sac was detected at the median lethal concentration.
• GC–MS analysis revealed the presence of sterols, fatty acids and some of its derivatives in the *n*-hexane fraction.

## Graphical abstract

**Figure:**
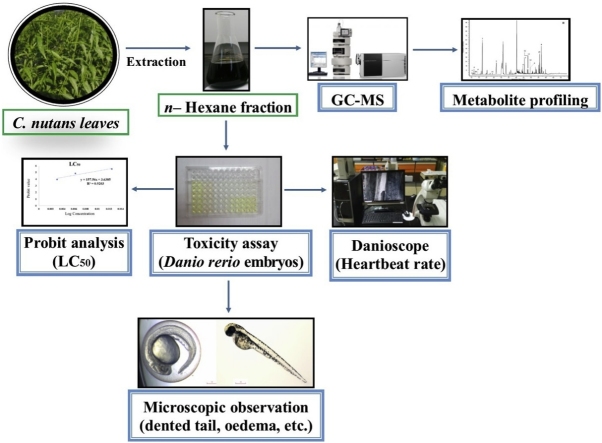

## 1 Introduction

Various medicinal plants and their constituents are reported to show profound pharmacological effects and widely applied for the treatment of various ailments. Extensive studies are carried out to evaluate the formulation and application of folk medicines with major concerns of their effectiveness and safety. Despite all their effective pharmacological functions, some of the phytoconstituents could to be potentially causing toxic, teratogenic, mutagenic, and carcinogenic as an individual compound or in combination(s) [1,2]. A teratogen is an agent that has the capability to induce or cause morphological abnormalities [1]. Therefore, studying the teratogenicity or toxic effect of medicinal plants using their potential extracts is crucial. Some of the common animal models used for toxicity studies include mammals such as mice, rats, rabbits and sheep, and for certain genetic and molecular studies, even flies and worms are used [3].

Zebrafish (*Danio rerio*), a tropical freshwater fish and a popular aquarium fish belonging to the minnow family (Cyprinidae) of the order Cypriniformes [4]. In recent times, zebrafish is emerging as a successful animal model, especially in *in vivo* drug discovery analysis carried out on their embryonic and larval stages can be performed easily and economically using multi-well plates. This species has been popular since the early 1970s as a valuable experimental model applied in studying vertebrate development, biological development and genetic illnesses. *D. rerio* is well-known for its regenerative abilities and found to be genetically complement to 70 % of human disease gene homologs, thus plays an important role as an essential tool for scientific research and as an ideal model for simulating pathological conditions in human [5].

As an animal model, the zebrafish embryo is a very popular and reliable tool due to its rapid developmental processes, transparency and high fecundity, low-maintenance in lab-scale, amenability to experimental manoeuvre and its similarity in embryonic development to vertebrates of higher forms [6]. The availability and reproduction of the zebrafish are reasonably high due to its ability to provide many non-adherent and transparent eggs under appropriate conditions. It is now being used for drug discovery and can be a useful and cost-effective alternative to some mammalian models [1].

*Clinacanthus nutans* (Burm. F) Lindau, locally famous as Sabah snake grass is a native Malaysian herb traditionally used as a functional food against gout, hyperuricaemia, inflammation, skin rashes, fever and diabetes [7]. *C. nutans* leaves have been traditionally used to treat skin rashes, snake bites and allergic reactions [7,8]. Previously, the lethal oral dose (LD_50_) of a polar extract (methanol extract) of this herb was measured in an acute oral toxicity study using mice and was reported to be more than 5000 mg/kg without observable adverse effects at doses greater than 2500 mg/kg [9]. However, the non-polar fraction of this herb has yet to be tested for its toxicity effects. Therefore, this study was intended to assess the toxicity effects and to determine the lethal concentration of the *C. nutans* leaf extract using zebrafish embryos. The teratogenicity effects of the extract were measured by the apparent clinical signs and symptoms [10].

## 2 Materials and methodology

### 2.1 Chemicals

The organic solvents (methanol and *n-*hexane) of analytical grade purchased from Merck (Darmstadt, Germany), while analytical grade dimethyl sulfoxide (DMSO) was from Fisher Scientific (Leicester, UK) was used. Pyridine and *N*-methyl-*N* (trimethylsilyl) trifluoroacetamide (97.0 %) were purchased from Sigma- Aldrich (St. Louis, MO, USA).

### 2.2 Plant material preparation

Plant material was harvested from an authorised farm named EES Biotech Sdn. Bhd at Bukit Mertajam, Penang, Malaysia. The whole plant sample was deposited at the Herbarium of the Natural Medicinal Product Centre at International Islamic University Malaysia (IIUM) located in Kuantan, Malaysia, for authentication. The registered specimen voucher number is PIIUM 0238-1. The fresh leaves were water washed and allow to dry at room temperature (25 ± 0.5 °C) under shade, for one week. The dried leaves were pulverised using a universal cutting mill (Fritsch, Germany) and kept in a −80 °C freezer for further process [11].

### 2.3 Fraction (*n-*hexane) preparation

Crude extract was obtained from 80 % methanol by macerating the coarsened *C. nutans* leaf powder in the solvent at the ratio of 1:3 (w/v) for 3 days, where the solvent was changed each consecutive day. The mixture was then filtered using Whatman filter paper No. 1 and the filtrate was evaporated using a rotary evaporator at 40 °C to recover the crude extract. The crude extract was then macerated in *n*-hexane at the ratio of 1:3 (w/v) to obtain the *n*-hexane fraction. The fraction was then filtered, evaporated and freeze dried to remove any remaining moisture before storage at −80 °C freezer before further analysis [11].

### 2.4 Toxicity assay

The toxic effect of the *C. nutans* leaf *n-*hexane fraction was analysed using a zebrafish embryo screening assay in a 96-well plate with prior maintenance and spawning procedures and care according to Organization for Economic Co-operation and Development (OECD) guideline [[12], [13], [14]].

### 2.5 Maintenance of zebrafish

The experiment using the zebrafish embryos, including the transportation and care of the animal was carried out according to the Institutional Animal Care & Use Committee - IIUM guidelines, with the registered approval number, IIUM/ IACUC Approval/ 2016/ (12) (85). The fish were obtained from a local pet store and maintained under a 10:14 h of dark:light cycle at a pH of 6.5–7.5 in an acrylic tank (9 L water capacity) occupied by 50 adult fish per tank. The tanks were kept in a closed multi-rack aquatic housing system (Aquaneering®, San Diego, California, USA) under laboratory conditions at a temperature of 27 ± 1 °C (optimum temperature: 26–28 °C) at the Central Research Animal Facility (CREAM) Zebrafish laboratory located at Kulliyyah of Pharmacy, IIUM, Kuantan. The water in the recirculating water system was aerated continuously with an aquarium air pump. The fish were fed twice per day with a Zeigler adult zebrafish complete diet composed of approximately crude protein (55 %), crude fibre (1.5 %), crude fat (15 %) and moisture (12 %) together with some ash and phosphorus [[14], [15], [16], [17], [18]].

### 2.6 Spawning of *Danio rerio* and embryo care

Healthy and active adult male and female zebrafish (ratio of 1:2) were selected and placed in a glass aquarium equipped with a continuous recirculation system under a 12:12 h of dark: light cycle. The water and air temperature were controlled at 27 ± 1 °C. Upon fertilization, the embryos were collected from spawn tanks, transferred into clean petri dishes containing embryo water prepared by dissolving 0.21 g of Instant Ocean® H salt in 1 L of Milli-Q water and allowed to grow until 6 hpf (hours post fertilization) [12]. The embryos were carefully washed to remove debris, while the unhealthy and dead embryos were removed by aspiration using a plastic pipette. Microscopic observation was performed at 6 hpf to observe the embryonic development prior to treatment [13].

### 2.7 Fraction preparation and treatment procedure

The fertilized healthy embryos were transferred into a 96-well plate with each well contained 1 embryo, and treatment was carried out using two sets of plates, with a set of control group on each plate. Each treatment group shall contain 20 embryos (10 embryos in each group x 2 plates), including the control group. The total volume of each well was 300 μL, consisting of 150 μL of embryo water aspirated with an embryo at 12 hpf and 150 μL of the plant fraction (H_fr_) prepared in 2 % DMSO, which yielded final concentrations of 15.63, 31.25, 62.5, 125, 250 and 500 μg/mL. A control group was prepared by exposing embryos to only 2% DMSO without sample. All plates were incubated at 27 ± 1 °C in a temperature-maintained room [13,14].

### 2.8 Microscopic observations

At 24, 48, 72 and 96 hpf, corresponding to 12, 36, 48 and 60 hpt (hours post treatment), respectively, the toxic effects of samples on zebrafish embryos were observed. The survival and sub-lethal endpoint were assessed during the treatment period. The sub-lethal endpoint evaluation measured include the quantification of hatching success and mortality rate, delayed developmental, frequency of oedema, and body anomalies [19].

The heartbeat rate was determined using DanioScope (version 1.1, Noldus Information Technology, Wageningen, The Netherlands), while the mortality (Eq. (1)) and hatchability (Eq. 2) rates were determined using the following equations:

(1) *Percentage of mortality (%) = No. of dead embryos/ Total embryos x 100*

(2) *Percentage of hatchability (%) = No. of hatched embryos/ Total embryos x 100*

Finally, the median lethal concentration (LC_50_) of the *C. nutans n-*hexane fraction was determined using probit analysis [17].

### 2.9 Metabolite profiling of the *n-*hexane fraction using GC–MS

A gas chromatography–mass spectroscopy (GC–MS) was used to identify the phytoconstituents present in the *n-*hexane fraction following a method described by Javadi et al. [20] with minor modification. The hexane fraction was derivatized before injection (1 μL) into the system in split mode (10:1). The Agilent 6890 GC–MS system used was equipped with an HP 5973 mass selective detector. A 5% phenyl methyl siloxane column (DB-5MS; Agilent Technologies, California, United States) of 250 × 0.25 μm (internal diameter and thickness respectively). The initial oven temperature was set at 190 °C, which was held for 30 min before raised to target temperature of 300 °C in 10 min (5 °C/min) and was finally increased to 320 °C in 10 min (10 °C/min). Helium was used as the carried gas with a flow rate of 1 mL/min. A full scan with a mass scan ranges from 50 to 550 *m/z* was acquired in monitoring mode. The mass spectra of the sample chromatogram peaks were compared with those in the in-house database library (NIST14). The chromatograms and mass spectra acquired were then processed using Agilent ChemStation and exported in MS format files. The raw data then converted into CDF format upon baseline correction using the Advanced Chemistry Development (ACD) laboratory software. XCMS software (Bioconductor version 2.9) using the R-guide program version 2.13.0 (R-Foundation for statistical computing) was applied to make chromatographic alignment and automatic peak detection, to ensure three-dimensional data table contain the arbitrary peak index (RT *m/z* pair), peak area (variables) and sample names (observations) at the end of analysis. This is followed by the conversion of data into a txt file and finally all information from the raw data extracted into a summarized Microsoft Excel file for further analysis with multivariate data analysis.

### 2.10 Statistical analysis

The data were represented as mean ± standard deviation (SD), with n = 20 using Minitab 17 (Minitab Inc., State College, Pa., USA). The *p* value was obtained from the ANOVA analysis using the Tukey’s test with *p* <  0.05 was considered significant. One-way ANOVA with a Tukey comparison test was used to evaluate the major differences between the samples with confidence interval of 95 %.

## 3 Results and discussion

### 3.1 Lethal concentration dose (LC_50_) of the *n-*hexane fraction

**Fig. 1:**
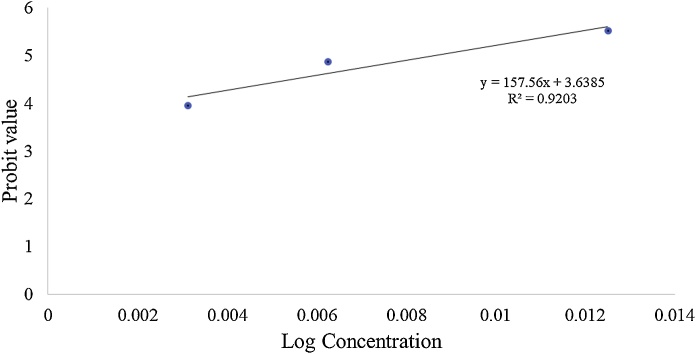
Median lethal concentration (LC_50_) value of the *C. nutans n-*hexane fraction based on probit analysis.

The LC_50_ value for H_fr_, which is indicated by the statistical estimation of the amount of toxicant (mg) per body weight (kg) required to induce the death of 50 % of the population of the animal tested, was calculated using probit analysis [17]. Fig. 1 displays the logarithmic estimation of the LC_50_ value using the concentration and mortality rate of the embryos. According to the OECD guideline [14], any toxicants are considered as harmful, toxic and highly toxic if the LC_50_ ranges between 10–100 mg/L, 1–10 mg/L and < 1 mg/L, respectively. Based on their scaling, the H_fr_ is considerably harmful with its LC_50_ calculated to be 75.49 mg/L.

### 3.2 Morphological defects in zebrafish embryos caused by the *C. nutans* leaf fraction

**Fig. 2:**
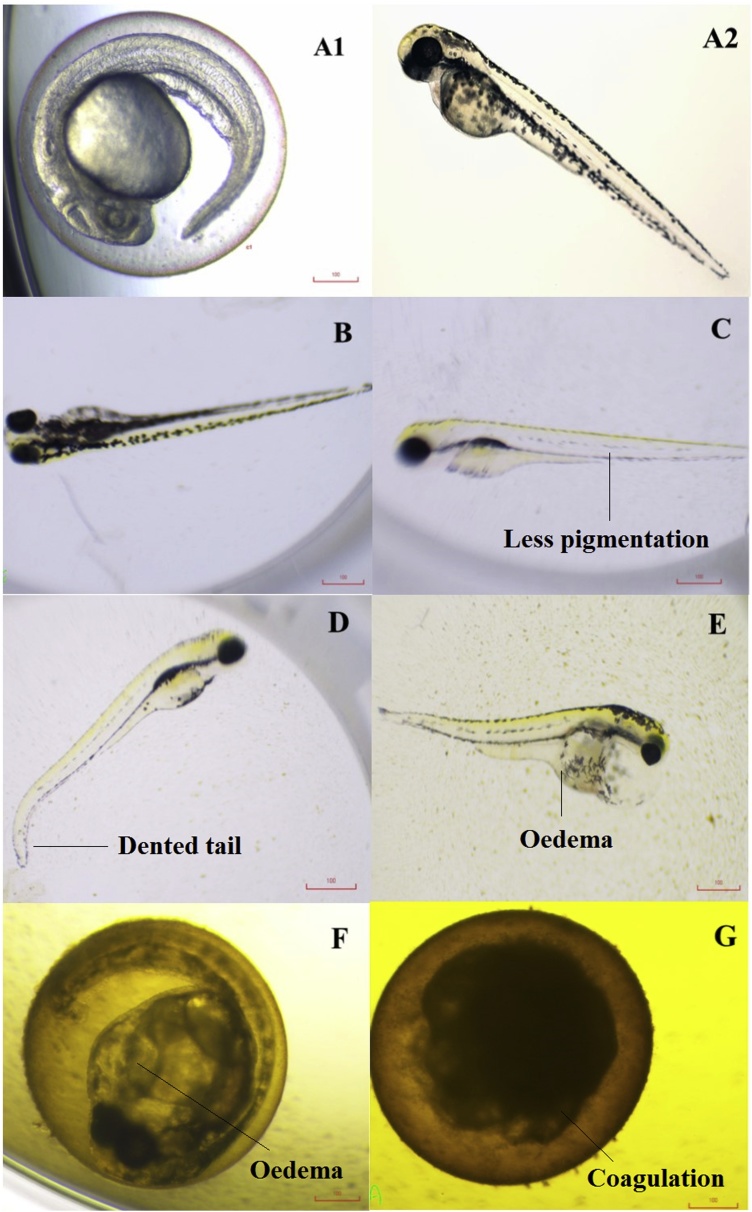
Morphological observation of *D. rerio* larvae treated with different concentrations of H_fr_ (A1: Normal embryo, A2: Normal larvae, B: 15.63 μg/mL, C: 31.25 μg/mL, D: 62.5 μg/mL E: 125 μg/mL, F: 250 μg/mL and G: 500 μg/mL) at 72 hpf and the defects observed (dented tail, less pigmentation, oedema and coagulation).

A control group and the treated groups, which were administered solvent (2% DMSO) and six different doses of the *n-*hexane fraction obtained from *C. nutans* leaves, respectively, were observed for toxic effects. The teratogenicity parameters (Table 1) and morphology (Fig. 2) were observed for both untreated embryos/larvae and those treated with different concentrations of H_fr_, viz., 15.63, 31.25, 62.5, 125, 250 and 500 μg/mL, at 72 hpf.

**Table 1:** Teratogenic defects of varying concentrations of *C. nutans* leaves extract at 72 hpf in *D. rerio* larvae.

Extracts Concentration (μg/mL)	Teratogenicity Parameters					
	Hyperactive	Delayed hatch	Crooked backbone	Less Pigmentation	Awkward position	Edema
15.63	–	–	–	–	–	–
31.25	–	**/**	–	–	–	–
62.5	**/**	**/**	–	–	–	–
125	**/**	**/**	**/**	**/**	**/**	**/**
250	nd	nd	nd	nd	nd	nd
500	nd	nd	nd	nd	nd	nd
Control	–	–	–	–	–	–

(-) - Not Detected; nd-not determined, (/)- present.

The observations made showed that all the larvae treated with the lowest plant concentration (B) had no obvious morphological defects, compared to the untreated group. The H_fr_ at 500 μg/mL was found to be toxic to the embryos, with all the embryos dying after 48 hpf, and some of embryos in these groups exhibited defects with crooked backbones and oedema. Whilst none of the embryos immersed in solution with the H_fr_ at 250 μg/mL survived after 72 hpf. Compared to the embryos in the control group, those immersed with plant fraction (H_fr_) at concentrations of 31.25 μg/mL and 62.5 μg/mL were observed to have less pigmentation and hyperactivity in movement, along with dented tail observed in only the latter group. Awkward positioning of the embryos was observed in the Fig. 2C, 2D and 2E. In the Fig. 2E group, the embryos showed defects with spinal curvature as well as oedema, and malformed yolk sacs along with body fluid accumulation that distorted the normal shape of the yolk mass were observed in the hatched larvae. Growth retardation is one of the important parameters of teratogenicity assays. Embryos in the untreated and lowest dose groups (15.63 μg/mL) showed the absence of the parameters mentioned above.

### 3.3 Mortality rate

Coagulation and the absence of heartbeat in zebrafish embryos are indicative of mortality [1]. Treatment with the lowest dose of the plant fraction (H_fr_) caused zero mortality towards the end of the study at 96 hpf. In contrast, the embryos treated with the highest dose of 500 μg/mL had an almost 50 % mortality rate at 24 hpf, and in the subsequent days of the treatment (48 hpf and 72 hpf), a 100 % mortality rate was observed. Coagulation can be seen in the embryos treated in 500 μg/mL of H_fr_, as shown in Fig. 2G. By contrast, the embryos immersed in the solution with an H_fr_ concentration of 250 μg/mL suffered from oedema and the absence of heartbeat (Fig. 2F).

### 3.4 Hatchability of zebrafish embryos

Hatching indicates the successful development of the embryo into larvae, which occurs between 48 and 72 hpf. However, as the concentration increased, the hatchability rate of H_fr_ treated embryos became significantly lower than the control groups’. Table 2 depicts the percent hatchability for the normal and the *C. nutans* leaf *n-*hexane fractions treated embryos at 72 hpf. The percent of hatchability of the embryos was observed to be normal and complete in the control group and in the group treated with 15.63 μg/mL of the plant fraction. The successful embryonic development of zebrafish embryos is indicated by hatching after 48 hpf [3]. Hatchability could not be measured in the embryos immersed in solution with the *n*-hexane fraction at concentrations of 250 μg/mL and 500 μg/mL due to the 75% mortality rate with coagulation before 48 and 24 hpf, respectively.

**Table 2:** The mortality rate, hatchability and heartbeat rate (72 hpf) of the developing embryos treated with different extract concentrations of *C. nutans* leaves hexane fraction (H_fr_) in *D. rerio* embryos.

Plant Extract Concentration (μg/mL)	Hatchability (%)	Mortality (%)	Heartbeat rate (bpm)/ Mean ± SD
16	100	0	140.14 ± 7.21^ab^
31	80	15	129.54 ± 5.83^b^
63	35	40	78.23 ± 12.17^c^
125	10	60	14.63 ± 4.10^d^
250	0	100	nd
500	0	100	nd
Control	100	0	152.42 ± 8.67^a^

The heartbeat rate data represented are in Mean ± Standard Deviation, n = 20.

Means that do not share a letter are significantly different (*p* < 0.05).

The hatchability and mortality are presented as percentages (%).

bpm- beats per minute, nd- Not determined.

### 3.5 Heartbeat rate

The normal heartbeat rate of zebrafish embryos ranges from 120 to 180 per min [21,22]. The mean heartbeat rates per minute (bpm) of zebrafish embryos exposed to varying concentrations of *C. nutans* H_fr_ at 72 hpf are presented in Table 2. The healthy embryos had the highest heartbeat rate of 152.42 per min, followed by the zebrafish treated with the fraction at concentrations of 15.63, 31.25, 62.5, and 125 μg/mL, having heartbeats of 140.14, 129.54, 78.23 and 14.63 bpm, respectively. No heartbeat rate was detected in the embryos immersed in solution with the plant fraction at concentrations of 250 μg/mL and 500 μg/mL due to early mortality.

### 3.6 Compound identification in the fraction (*n-*hexane)

All the metabolites identified in the *n-*hexane fraction were confirmed by analysing and comparing the spectral pattern with the GC–MS database library (NIST14) [20]. The constituents identified majorly belongs to the sterols, organic acids, fatty acids, and others, namely phytol, stigmasterol, 1-monopalmitin, heptadecanoic acid, palmitic acid, hexadecanoic acid, pentadecanoic acid, 1-linolenoylglycerol and stigmast-5-ene, with a similarity index of more than 90%. All the compounds identified are tabulated in Table 3 along with their similarity indices.

**Table 3:** Compounds identified in the *C. nutans* leaves hexane fraction using GC–MS.

No.	Compounds	RT (min)	% of Area	Probability	M^+^	Molecular Formula
1	Palmitic acid	17.18	10.89	99	256.24	C_16_H_32_O_2_
2	Phytol	26.59	5.51	95	296.31	C_20_H_40_O
3	Hexadecanoic acid	11.60	0.97	99	270.26	C_17_H_34_O_2_
4	1- Monopalmitin	44.44	1.00	95	330.51	C_19_H_38_O_4_
5	Stigmast-5-ene	56.83	4.74	99	398.39	C_29_H_50_
6	Pentadecanoic acid	12.31	0.26	98	242.22	C_15_H_30_O_2_
7	Heptadecanoic acid	23.90	0.15	99	270.26	C_17_H_34_O_2_
8	1-Linolenoylglycerol	47.60	0.51	99	352.52	C_17_H_36_O
9	Stigmasterol	55.61	2.90	99	412.37	C_29_H_48_O

RT = Retention Time.

C = Carbon, H=Hydrogen, O = Oxygen, N = Nitrogen.

## 4 Discussion

The LC_50_ value of this plant fraction to the zebrafish embryo reveals that it is harmful to zebrafish embryos. Some morphological changes were observed in the zebrafish treated with the plant fraction at concentrations near its LC_50_ value, as described in the results section. In this study, embryos immersed in various concentrations of the H_fr_ displayed several morphological changes with defects during development in a concentration and time of exposure-dependent style [23]. The developing embryos in their early juvenile stages are among the most delicate [6].

Apparently, the hatching rate of the embryos are affected by the varying concentration of the plant fraction, where, as the sample concentration increased, the hatchability rate decreased. The low hatchability rate and delayed hatching process indicate growth retardation. The delayed hatching could be due to developmental abnormalities in the evolving embryos, resulting in the inability of the chorion to break; it also could be explained by the morphological abnormalities observed in the embryos, which limit hatching [24].

Besides that, hatchability in higher sample concentrations may have been affected by the weakened of the chorionic membrane or due to the induced chorionase enzyme activity [13]. Under the embryonic conditions and increasing sample concentrations, oedema formation emerges and observed to persist even after hatching. As the fraction concentration increases from 125 to 250 μg/mL, the oedema size increases significantly which is possibly caused by the osmoregulatory system failure associated with toxicants build-up [13].

Another obvious and common deformity observed in the groups treated with higher concentrations of H_fr_ was spinal curvature. Three types of spinal curvatures namely kyphosis, lordosis and scoliosis, that are measured based on the spinal bending degree depend on few factors including accumulation of different toxicants, AChE enzyme inhibition and neuromuscular coordination deficit. The literature further indicates that spinal deformities may occur due to reduced collagen level in the spinal column, alteration in the amino acid composition or inhibition/downregulation of specific gene regulator, protein tyrosine kinase 7 (ptk7) gene that functions to be an important regulator of Wnt signalling [13,25].

P’ng et al. [26] conducted a cytotoxicity study using a polar methanolic extract of *C. nutans* that showed significant cytotoxicity at 200 and 100 μg/mL to all tested cell lines. Based on their study, the methanolic extract of *C. nutans* showed significant cytotoxicity at 200 and 100 μg/mL. The percentage of inhibition at the concentration of 100 μg/mL and 200 μg/mL was 93 ± 1.0 % and 84 ± 1.1 %, respectively.

Plant metabolites have multifunction, where the secondary metabolites are produced for the survival and “housekeeping” of the organism. These metabolites may exert therapeutic as well toxic effects on human beings [27,28]. GC—MS profiling of the *n-*hexane fraction exhibited the presence various plant metabolites. Terpenoids, which are produced by plants to function as phytoalexins in the defence system or even as signals in defence responses to external defoliators, feeders, predators and parasitoids, are known to be a structurally diverse group. Some of the volatile terpenoids with a strong bitter taste, especially toxic terpenes, are meant to expel certain animals and thus function as antifeedants. Previous studies have reported that the presence of phytosterols such as stigmasterol, β-sitosterol and lupeol in the *n-*hexane fraction of *C. nutans* may contribute to this toxic effect [[28], [29], [30]]. This finding agrees with the results of GC—MS result showed the presence of stigmasterol as one of the major constituents. Phytosterols are known to exert cytotoxic effects in combination with other bioactive compounds, and the antagonistic or synergistic interaction of the phytoconstituents may be responsible for causing the toxic effects of the plant fraction to the embryos [7]. Generally, non-polar compounds possesses much lower molecular weight comparably, which allows them to pass through the blood brain barrier thus, are capable of inducing various deformities including inhibition of AChE and other neurological damages [31].

In a study done by Arullapan et al. [8] indicated the cytotoxicity effects of *C. nutans* leaves extract on cancer cell line (HeLa and K-562 cells) causing reduced proliferation effect and fluctuation in the viability as the extract concentration increased after 72 h incubation. The extract contained phytol, stigmasterol, and fatty acids including hexadecanoic and pentadecanoic acids that are comparable to the findings of this study.

Besides stigmasterol, palmitic acid was also found in abundance in the extract. The presence of saturated fatty acids especially palmitic acid was reported to induce apoptosis. In a study using pancreatic cells, palmitic acid was found to cause apoptosis via mtDNA and genomic DNA damages, and it was also reported to have induced cell death by down-regulating cardiolipin, a phospholipid and an important component of the inner mitochondria membrane which has multiple role in various cellular processes inside and outside of the mitochondria [32].

In addition, the presence of cardiac glycosides in the plant extract may also cause abnormalities in cardiological functions [13,33]. Technically, some other toxic compounds that have yet to be identified may also contribute to this effect on zebrafish embryos. Compounds exerting toxicity effects in the *C. nutans* leaves fraction should be further identified and removed from the fraction to enable the beneficial bioactive compounds to be able to exert their bioactivities without any interference.

## 5 Conclusion

This study revealed that the toxic effects of the *C. nutans* leaf *n-*hexane fraction (H_fr_) on developing zebrafish embryos are dependent on the time of exposure to and concentrations of the extract. At 72 hpf, the median lethal concentration (LC_50_) of *C. nutans* H_fr_ was determined to be 75.49 μg/mL, with obvious morphological defects such as less pigmentation, dented tail, spinal curvature, oedema malformed yolk sac as well as reduced hatchability and growth retardation, thus indicating it is harmful. There is still a need for further research in order to understand and explain the specific effects *C. nutans* metabolites and to draw conclusions regarding human risk assessment.

## Declaration of Competing Interest

There are no conflicts to declare.
